# Induced Resistance by Ascorbate Oxidation Involves Potentiating of the Phenylpropanoid Pathway and Improved Rice Tolerance to Parasitic Nematodes

**DOI:** 10.3389/fpls.2021.713870

**Published:** 2021-08-11

**Authors:** Richard Raj Singh, Jessil Ann Pajar, Kris Audenaert, Tina Kyndt

**Affiliations:** ^1^Department of Biotechnology, Faculty of Bioscience Engineering, Ghent University, Ghent, Belgium; ^2^Department of Plants and Crops, Ghent University, Ghent, Belgium

**Keywords:** tolerance, priming, phenotyping, *Oryza sativa*, *Meloidogyne graminicola*, phenylalanine ammonia lyase

## Abstract

Anticipating an increased ecological awareness, scientists have been exploring new strategies to reduce the use of chemical pesticides to control pests and diseases. Triggering the intrinsic plant defense system is one of the promising strategies to reduce yield loss by pathogenic organisms, such as nematodes. Ascorbate oxidase (AO) enzyme plays an important role in plant defense by regulating the apoplastic ascorbate/dehydroascorbate (DHA) ratio *via* the ascorbate oxidation process. Ascorbate oxidation is known to induce systemic resistance in rice against parasitic root-knot nematodes (RKN). Here, we sought to evaluate if AO- or DHA-induced resistance (IR) against RKN *M. graminicola* involves activation of the phenylpropanoid pathway and whether this IR phenotype has potential effects on growth of rice seedlings under stressed and unstressed conditions. Our results show that AO/DHA-IR against these parasitic nematodes is dependent on activation of phenylalanine ammonia lyase (PAL). However, application of reduced ascorbic acid (AA) did not induce this response. Gene expression analysis *via* qRT-PCR showed that *OsPAL2* and *OsPAL4* are highly expressed in AO/DHA-sprayed nematode-infected roots and PAL-activity measurements confirmed that AO/DHA spraying triggers the plants for primed activation of this enzyme upon nematode infection. AO/DHA-IR is not effective in plants sprayed with a chemical PAL inhibitor confirming that AO/DHA-induced resistance is dependent on PAL activity. Improved plant growth and low nematode infection in AO/DHA-sprayed plants was found to be correlated with an increase in shoot chlorophyll fluorescence (Fv/Fm), chlorophyll index (ChlIdx), and modified anthocyanin reflection index which were proven to be good above-ground parameters for nematode infestation. A detailed growth analysis confirmed the improved growth of AO/DHA-treated plants under nematode-infected conditions. Taken together, our results indicate that ascorbate oxidation enhances the phenylpropanoid-based response to nematode infection and leads to a tolerance phenotype in treated rice plants.

## Introduction

Rice (*Oryza sativa* L.) is one of the world’s most valuable agricultural commodities, a staple food for half of the global population, and an important model plant for the study of the interaction between a monocotyledonous plant and plant parasitic nematodes at molecular and physiological level (De Waele and Elsen, 2007; Kyndt et al., 2014). Root-knot nematode (RKN) *Meloidogyne graminicola* is an obligate endo-parasitic root pathogen (Mantelin et al., 2017), with significant detrimental effects on rice (Bridge et al., 2005; De Waele and Elsen, 2007). *M. graminicola* is dominant in rainfed and lowland (irrigated) rice, as well as in deep-water ecosystems (Prot and Rahman, 1994). It is known to cause substantial yield losses in all the rice growing belts of southeast Asia (Rao and Biswas, 1973; Kumari et al., 2016). An estimated yield loss caused by this nematode can be up to 87% of production (Plowright and Bridge, 1990; Netscher, 1993). *M. graminicola* has a short life cycle when compared with other RKN species: 19–27 days at temperatures of 22–29°C (Bridge and Page, 1982). Its short life cycle and wide host range make this species difficult to control (De Waele and Elsen, 2007). The infective second-stage juvenile (J2) penetrates the rice root in the elongation zone, migrates to the vascular tissue, and forms a feeding site, consisting of 3–8 giant cells (Kyndt et al., 2014). These giant cells function as a specialized sink, supplying nutrients to the nematode for its development and reproduction (Caillaud et al., 2008). Female nematodes will develop within 14 days after infection, after which they start to lay eggs (Kyndt et al., 2014). The females lay hundreds of eggs in a protective gelatinous matrix, forming an egg mass. *M. graminicola* females lay eggs in the root cortex embedded in galls while in other RKN species egg masses are found on the root surface (Kyndt et al., 2014; Escobar et al., 2015). Hyperplasia and hypertrophy of the surrounding cells lead to the development of root knots (galls) and hooked root tips, typical root symptoms induced by *M. graminicola* infection (Bridge et al., 2005; Karssen et al., 2006; Karssen and Moens, 2006; Kyndt et al., 2014). This gall formation disturbs normal root physiological functions, such as water and nutrient transport, ultimately causing chlorosis and stunted growth and observable patchy growth in rice fields (Bridge and Page, 1982).

Induced resistance (IR) is an enhanced plant disease resistance phenotype in response to stimulation by a pathogen, insect herbivory, wounding, a beneficial microbe, or by exogenous application of natural or synthetic compounds (Conrath, 2006; Bektas and Eulgem, 2015; Conrath et al., 2015). IR can work through direct activation of plant defense even without pathogen challenge and/or it can work *via* defense priming whereby some genes, enzymes, or pathways are only activated when the plant is challenged by a pathogen (De Kesel et al., 2021). Upon this challenge, the plant effectively mounts a faster and/or stronger defense response resulting in reduced disease and/or stress tolerance (Mauch-Mani et al., 2017). However, potential changes in plant development and plant growth should be monitored upon IR activation, as excessive activation of plant defense can have fitness costs (van Hulten et al., 2006; Wu et al., 2010; Koen et al., 2014; Luna et al., 2014, 2020; Cohen et al., 2016; Yassin et al., 2021).

Compounds, such as beta-aminobutyric acid (BABA; Ji et al., 2015), thiamine (Huang et al., 2016), silicon (Zhan et al., 2018), methyl jasmonate (MeJA), ethephon (Eth), the salicylic acid (SA) analogue benzothiadiazole BTH (Nahar et al., 2011), COS-OGA (Singh et al., 2019), and ascorbic acid (AA, vitamin C; Arrigoni et al., 1979; Al-Sayed and Thomason, 1988; Osman, 1993; Hamada et al., 2000; Osman et al., 2013), are known to induce resistance against RKN. AA is a water-soluble compound and the most abundant antioxidant in plants (Smirnoff, 2018; Foyer et al., 2020). The redox status of total AA in the apoplast, regulated by ascorbate oxidase (AO; Pignocchi and Foyer, 2003; Foyer and Noctor, 2005; Fotopoulos and Kanellis, 2013), is known to influence plant growth. This is mediated by effect on hormone pathways, antioxidant enzyme activities, mitogen-activated protein kinase (MAPK) activity, and calcium channels (Pignocchi and Foyer, 2003). AO catalyzes oxidation of AA to the unstable radical monodehydroascorbate (MDHA) and subsequently to DHA in the apoplast. DHA is then transported to the symplast and reduced back to AA through the symplastic AA–glutathione (AA–GSH) cycle (Horemans et al., 2000), which includes among others dehydroascorbate reductases (Smirnoff, 2000; Smirnoff and Wheeler, 2000).

Secondary metabolites play an important role in plant defense against parasitic nematodes (Desmedt et al., 2020). One of the best-known secondary metabolic pathways in plants is the phenylpropanoid pathway. Phenylpropanoids are derived from cinnamic acid, which is formed from phenylalanine (Vogt, 2010). The phenylpropanoid pathway is constituted of a complex series of branching biochemical pathways. This plant-specific pathway produces a variety of compounds, including structural cell wall components (lignin, suberin, and other cell wall-associated phenolics), antioxidants (flavonoids and anthocyanins), immunity signals (SA), and toxins (coumarins and furanocoumarins; Dixon et al., 2002; Vogt, 2010; Lefevere et al., 2020). The first step in the phenylpropanoid pathway, where phenylalanine ammonia lyase (PAL) catalyzes the conversion of phenylalanine to trans-cinnamate, is important in the transition between primary and secondary metabolism (Hahlbrock and Scheel, 1989; Dixon and Paiva, 1995; Huang et al., 2010; Vogt, 2010). The expression of the *PAL* gene responds to biotic and abiotic stresses, such as pathogens, UV irradiation, and low temperature (Dixon and Paiva, 1995; MacDonald and D’Cunha, 2007). PAL can be induced by wounding and insect herbivory, pathogen infection, and abiotic stresses (Hahlbrock and Scheel, 1989; Dixon and Paiva, 1995), as well as by jasmonate (Taheri and Tarighi, 2010, 2011).

In previous research (Singh et al., 2020b), we have shown that ascorbate oxidation by exogenous application of AO induces rice defense against *M. graminicola*. The process involves systemic activation of the ethylene/jasmonate pathway coupled with high H_2_O_2_ accumulation upon nematode infection. We also confirmed that exogenous application of the product of AO, namely, DHA, gives a similar IR phenotype. This uncovered a previously unknown role for ascorbate oxidation in activation of systemic resistance pathways against nematodes. In this research, we sought to evaluate whether IR induced by ascorbate oxidation involves activation of the phenylpropanoid pathway and whether this IR phenotype has plant growth effects. After confirmation of the DHA and AO-IR phenotype against RKN in rice, we analyzed the expression of *OsPAL2* and *OsPAL4* and quantified PAL activity in the shoots and roots of DHA/AO-treated plants, both under uninfected and nematode-infected conditions. Then, we manipulated PAL levels by treating plants with the PAL inhibitor, L-2-aminooxy-3-phenylpropanoic acid (AOPP) and analyzed the susceptibility of rice plants to nematodes. To study the morphological and physiological consequences of the AO/DHA application, a state-of-the-art high-throughput sensor-to-plant phenotyping platform (automated phenotyping platform, APP) was used. We used image analysis to quantify the changes in plant physiology of rice upon AO/DHA application, with and without nematode infection. Finally, the growth of AO- or DHA-treated rice plants was monitored to evaluate potential trade-off effects of the IR phenotype.

## Materials and Methods

### Plant Material and Growth Conditions

Rice (*Oryza sativa* subsp. *japonica*) seeds of cultivar Nipponbare were provided by the US Department of Agriculture (GSOR-100). Germination was set by placing seeds on wet filter paper in a petri dish, which was then incubated at 30°C for 4 days. Thereafter, the seedlings were transplanted in polyvinyl-chloride tubes (one seedling per tube) containing a mixture of sterilized fine sand and synthetic absorbent polymer (SAP) substrate (Nahar et al., 2011; Huang et al., 2016). Rice seedlings were further kept in a growth room at 26°C, 12 h/12 h light regime (150 μmol/m^2^s) and relative humidity of 70–75%. The plants were fertilized by giving 10 ml of Hoagland solution three times a week.

### Plant Treatments

The following chemicals with respective concentrations were used as: reduced ascorbic acid (AA; Sigma-Aldrich, Missouri, United States) at 20 mM (Singh et al., 2020b), ascorbate oxidase (AO; Sigma-Aldrich, Missouri, United States) at 20 U/ml (Singh et al., 2020b), dehydroascorbic acid (DHA, Sigma-Aldrich, Missouri, United States) at 20 mM (Singh et al., 2020b), methyl jasmonate (MeJA, Sigma-Aldrich) at 100 μM (Nahar et al., 2011), and AOPP, an inhibitor of PAL activity, at 100 μM (Ji et al., 2015; Khanam et al., 2018). These concentrations have been optimized in the previous publications with chemical concentrations tested for bio-efficacy and lack of phytotoxicity. AOPP was dissolved in 1 ml of EtOH before diluting further in water. All other chemicals were dissolved in water. To allow efficient uptake, all solutions were supplemented with 0.02% (v/v) of Tween20 (Nahar et al., 2011) and were then administered *via* foliar spraying using Fantasea spray bottles (Jojoli, Netherlands). This allows spraying a fine mist without clogging. In each experiment, 14-day-old plants were sprayed with 6.25 ml of solution until runoff. Foliar spraying was done 24 h prior to nematode or mock inoculation. Plants in the control group were mock sprayed with distilled water containing 0.02% (v/v) of Tween20.

### Nematode Inoculation and Analysis of Nematode Infection

A pure culture of *M. graminicola* originating from the Philippines (kindly provided by Prof. D. De Waele, Catholic University, Leuven, Belgium) was maintained on *O. sativa* cv. Nipponbare plants grown in potting soil under conditions similar to the experimental plants, as described earlier. After 3 months of infection, second-stage juveniles (J2) were extracted following the modified Baermann funnel method (Bridge et al., 2005) and were used as inoculum. The roots of rice host plants were washed thoroughly under running water. The roots consisting of galls were finely chopped (5 mm). The *M. graminicola* J2 suspension was collected 48 h after the extraction and was concentrated using centrifugation for 10 min at 1,500 rpm at room temperature. One day after treatment, 15-day-old plants were inoculated with approximately (≈)250 J2 of *M. graminicola* or mock inoculated with water. Plant susceptibility was evaluated at 14 days after inoculation (dai) by counting the number of galls. In addition, nematode reproduction was assessed by counting the number of egg-laying females (ELFs) per plant. To count the number of galls and ELFs, the plant roots were soaked in a boiling acid fuchsin solution for 3 min (0.8% acetic acid and 0.013% acid fuchsin; Nahar et al., 2011). Roots were then rinsed with running tap water to remove excess acid fuchsin and destained in acid glycerol. Galls and ELFs were counted using a stereomicroscope. All nematode infection experiments were repeated at least twice, each time including eight plants per treatment. The experimental timeline for spraying, nematode inoculation, and evaluating nematode susceptibility is shown in Supplementary Figure 1A.

### Evaluation of Physiological Effects of DHA- and AO-Induced Resistance

To evaluate direct activation and/or a primed defense response, we set up a multifactorial experiment including eight groups of plants: (1) naïve plants uninfected (Ctrl), (2) naïve infected plants, (3) AA-sprayed uninfected, (4) AA-sprayed infected, (5) AO-sprayed uninfected, (6) AO-sprayed infected, (7) DHA-sprayed uninfected, and (8) DHA-sprayed infected plants. In all cases, 14-day-old plants were sprayed with chemicals or mock sprayed with water including 0.02% Tween20. Twenty-four hours later, plants were either mock inoculated or inoculated with ≈250 J2 nematodes. Evaluation of different parameters (see below) was done at 3 dai. The experimental timeline for spraying, nematode inoculation, and evaluation of different parameters (sampling) is shown in Supplementary Figure 1B.

#### PAL-Activity Measurement

Four biological replicates per treatment were sampled, with each biological replicate containing samples/tissues from 4–5 plants. Samples included galls, root tips (of uninfected plants), and complete shoots of infected or uninfected plants. After immediate freezing in liquid nitrogen (N_2_) and grinding PAL activity was measured according to Camacho-Cristóbal et al. (2002). One hundred mg of each sample was dissolved in 800 μl of 50 mm sodium phosphate buffer containing 2% (w/v) poly-vinylpolypyrrolidone, 2 mm EDTA, 18 mm-mercaptoethanol, and 0.1% (v/v) Triton X-100. The homogenate was centrifuged at 8,000 rpm, at 4°C for 10 min. One hundred and thirty-five μl of reaction buffer was mixed with 50 μl of 5 mm of L-phenylalanine, and 20 μl of supernatant in separate tubes, after which absorbance was measured at 290 nm. Subsequently, the sample was incubated for 30 min at 40°C in a water bath, after which 10 μl of hydrochloric acid was added and mixed for 10 min. PAL activity was assayed by measuring the formation of trans-cinnamic acid at 290 nm, where 1 unit (U) of PAL activity is the amount of enzyme that produced 1 nmol trans-cinnamic acid per hour. Negative control reactions had no L-phenylalanine as substrate.

#### qRT-PCR

RNA was extracted using the Plant RNeasy Plant Mini kit (Qiagen) following the manufacturer’s instructions. For each treatment, three biological replicates were sampled and analyzed, each consisting of a pool of root or shoot tissue of at least 4–5 plants per treatment. qRT-PCR was performed and analyzed as described in Huang et al. (2015), using normalization based on three reference genes, *OsEIF5C* (LOC_Os11g21990), *OsEXP* (LOC_Os03g27010), and *OsEXPNarcai* (LOC_Os07g02340). Primer pairs for *OsPAL2* and *OsPAL4* are described in Tonnessen et al. (2015) and for the reference genes in Huang et al. (2015).

#### Spectral Phenotyping and Image Analysis

The morphological and physiological changes in rice leaves were monitored using an APP, which visualizes diverse physiological traits in real time, based on specific absorption, reflection, and fluorescence patterns in visible and near-infrared (NIR) wavelengths. The platform consists of a 3CCD 6 Mp—16 bit camera mounted on a Cartesian coordinate robot, equipped with 12 optical interference filters (CropReporter, PhenoVation B.V., Wageningen, Netherlands). A total of five plants per treatment were monitored. The camera captured following images: RGB (red green blue) images, reflectance spectra to calculate the anthocyanin index and chlorophyll index (ChlIdx) and the minimal fluorescence, F_0_, and the maximum fluorescence, F_m_. Images were processed *via* the “Data Analysis Software” program (PhenoVation B.V., Wageningen, Netherlands).

The modified anthocyanin reflectance index (mARI) was calculated using following formula (Gitelson et al., 2009):

$$\mathrm{mARI}=\left(\frac{1}{\rho_{\;550\mathrm{nm}}}-\frac{1}{\rho_{\;710\mathrm{nm}}}\right)\rho_{770nm}$$

The ChlIdx was determined using following formula (Gitelson et al., 2009):

$$\mathrm{ChlIdx}=\left(\frac{\rho_{770nm}}{\rho_{\;710\mathrm{nm}}}-1\right)$$

where ρ_550_ is the reflectance in the first spectral band, which is maximally sensitive to anthocyanin content; ρ_710_ the reflectance in the second spectral band, which is maximally sensitive to chlorophyll content but not sensitive to anthocyanin content; and ρ_770_ the reflectance of the third spectral band, which compensates for leaf thickness and density.

The maximum quantum efficiency of photosystem II (F_v_/F_m_) was calculated using the formula of Baker (2008):

$$F_{v}/F_{m}=\left(F_{m}-F_{0}\right)/F_{m}$$

Before measurement, the plants were placed in the dark for 30 min to allow for dark adaptation of the leaves to maximally oxidize primary quinone acceptor QA. The minimal (F0) and maximal fluorescence (Fm) were quantified based on the OJIP induction curve according to the manufacturers specifications (Björkman and Demmig, 1987).

### Evaluation of Growth Parameters of Treated Plants Under Nematode-Infected and Uninfected Conditions

The assessment of plant growth rate upon application of a plant protection product is an essential element in its efficacy evaluation (Kalamarakis and Markellou, 2007). To evaluate the plant growth rate in response to AA, AO, or DHA treatment and this under unstressed or nematode-uninfected conditions, a multifactorial experiment with eight groups of plants (see above) was set up and evaluation was done at different time points. Seed germination and growth in SAP were performed as described above. The shoot height of the 14-days old plants was recorded, after which rice plants were sprayed with the respective chemicals or mock sprayed with water as explained above. One day (24 h) after the treatment, the rice plants were inoculated with *M. graminicola* J2s (Mg^+^) or mock inoculated with water (Mg^−^). To evaluate the shoot growth rate, shoot height was measured at three time points 18, 22, and 26 days old on the same group of plants. To assess root growth, however, three independent groups of plants had to be set-up for end-point evaluation of root length, each at another time point (18, 22, or 26 days old). This was required because the trauma by uprooting from the substrate causes plant damage, potentially preventing re-establishment and affecting the plant growth rate. For all experiments, eight plants were used per treatment and all experiments were three times independently repeated. The shoot height and root length was measured with a ruler, taking the distance between the soil surface and the root apical meristem or the longest leaf of the plant.

## Results

### Foliar Spraying With AO and DHA Induces Systemic Resistance in Rice Roots Against *M. graminicola* Infection and Promotes Growth

**Figure 1:**
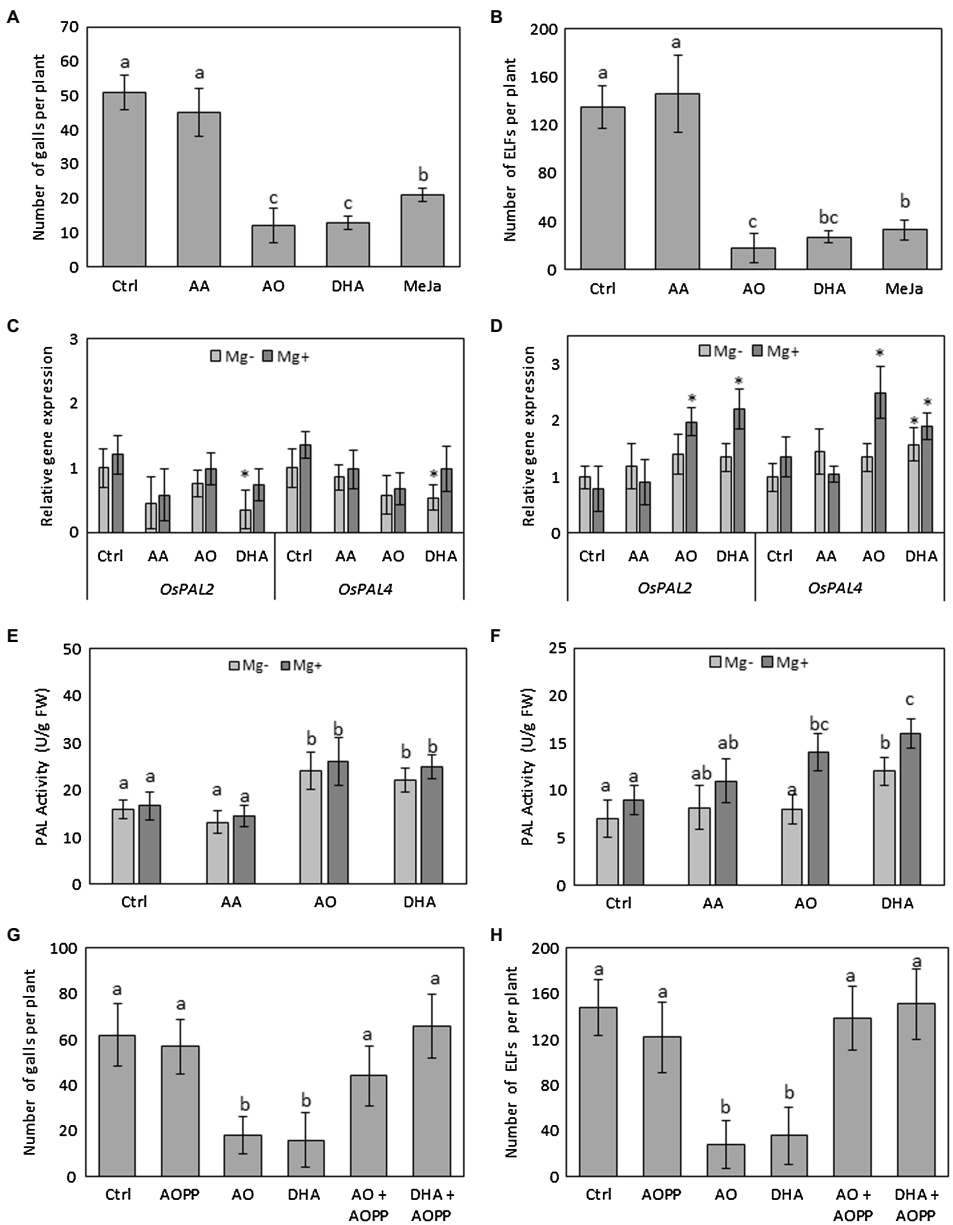
Foliar spraying with AO and DHA induces systemic resistance in rice roots against *M. graminicola* through activation of PAL. Fourteen-day-old wild-type Nipponbare rice plants were sprayed with 20 mm of AA, 20 ml/unit of AO, and 20 mm of DHA or mock sprayed (Ctrl). After 24 h, plants from each treatment were inoculated with ≈250 s-stage juveniles of *M. graminicola* per plant. Total number of **(A)** galls and **(B)** egg-laying females (ELF) at 14 dai. **(C,D)** relative expression levels of phenylalanine ammonia lyase genes in shoot and root tissue at 3dai (*n* = 3). **(E,F)** PAL enzyme activity in shoots or nematode-induced galls of infected and uninfected plants at 3 dai (*n* = 4). (**G,H**) total numbers of galls and ELFs in L-2-aminooxy-3-phenylpropanoic acid (AOPP)-sprayed plants with and without DHA or AO treatment. For nematode infection experiments **(A,B, G,H)**, data represent mean ±SD (*n* = 8) and data were analyzed by student *t-test* at *p ≤* 0.05. For **C,D**, gene expression levels were normalized using three internal reference genes *OsEIF5C*, *OsEXP*, and *OsEXPNarcai*. Data are shown as relative transcript levels in comparison with the control plants (expression level set at 1). In c and d asterisks indicate significant differential expression between AO-treated and control plants (REST-analysis; *α* = 0.05). For **E,F**, data were analyzed by one-way ANOVA followed by Tukey’s *post-hoc* test. Different letters indicate statistically different means at *p ≤* 0.05.

Previously, we evaluated the effect of the compounds (AA, AO, and DHA) on the number of galls and number of nematodes and a significantly lower number of galls and nematodes was observed in rice plants sprayed with AO or with DHA, but not with AA (Singh et al., 2020b). Here, the effect of foliar AA, AO, or DHA treatment on rice plants was independently confirmed by counting the number of galls and extended by counting the total number of egg-laying females (ELFs). Reproduction capacity of a nematode is indicated by the number of ELFs and resistant plants support low or no nematode reproduction (Cook, 1987; Roberts, 2002). Foliar application of AO and DHA on rice plants significantly reduced the number of galls recorded at 14 days after inoculation (dai; 76 and 74% reduction, respectively; Figure 1A, Supplementary Figure 1C) when compared with mock-sprayed plants, confirming our previous results (Singh et al., 2020b). Furthermore, spraying with AO and DHA also reduced the number of ELFs (87 and 80% reduction, respectively; Figure 1B, Supplementary Figure 1C). Spraying with AA did not cause any significant changes in the number of galls (Figure 1A) or ELFs (Figure 1B). Application of positive control methyl jasmonate (MeJA) also significantly reduced gall and ELF development, consistent with the data shown before (Nahar et al., 2011; Verbeek et al., 2019).

To evaluate potential fitness costs of this IR phenotype, different plant growth parameters were compared between AA/AO/DHA-sprayed and mock-sprayed plants at the end of this experiment (14 dai). Foliar application of AO and DHA resulted in a significant increase (19 and 20%, respectively) in shoot height and root length (25 and 44%) and fresh root weight (34 and 49%) in comparison with that of mock-sprayed plants (Supplementary Figures 2A–D). No effect of AA on plant growth (shoot height, root length, and root weight) was observed (Supplementary Figure 2). These results reveal that AA, AO, and DHA pose no negative effects on plant growth and apparent morphology at 15 days after treatment. In fact, pre-treatment with 20 U/ml AO or 20 mm of DHA seemed to improve the shoot and root growth of plants compared to that of mock-sprayed and AA-sprayed plants.

### AO/DHA-Induced Resistance Against Nematodes Is Dependent on Activation of PAL

To investigate whether the low *M. graminicola* susceptibility in AO- and DHA-sprayed rice plants was due to alterations in the phenylpropanoid pathway, 14-day-old rice plants were sprayed with AO or DHA or mock-sprayed. After 24 h, a subset of plants was inoculated with nematodes (Mg^+^) while another subset was mock inoculated with water (Mg^−^). We quantified the expression of Os*PAL2* and *OsPAL4* and measured the PAL activity at 3 days after inoculation, which corresponds with 4 days after spraying. Analyses were done on shoots of infected and non-infected plants as well as on galls versus root tips for uninfected plants. Our qRT-PCR analyses show that both *OsPAL2* and *OsPAL4* are significantly downregulated in the shoots of DHA-sprayed uninfected plants when compared with uninfected untreated plants (Figure 1C). However, in the root tissues, the expression of the *OsPAL4* gene was significantly induced upon DHA spraying (Figure 1D). Interestingly in the nematode-induced galls, significantly higher expression levels of *OsPAL2* and *OsPAL4* genes were observed in both AO as well as DHA-sprayed plants when compared with mock-sprayed uninfected or infected plants (Figure 1D).

PAL-activity measurements confirmed its enzymatic activation. Data show significant increase (~32%) in PAL activity in the shoots of AO/DHA-sprayed plants when compared with mock-sprayed plants, regardless of the infection (Figure 1E). Moreover, in galls of AO/DHA-sprayed plants, a significant increase in PAL activity was observed when compared with uninfected root tips of AO/DHA-sprayed plants (Figure 1F), suggesting that AO/DHA spraying triggers the plants for primed PAL activity in galls upon nematode infection.

To confirm the involvement of PAL activity in AO/DHA-induced resistance, an independent infection experiment was carried out using foliar application of a chemical PAL inhibitor AOPP. When chemically blocking this enzyme, no significant differences were observed in number of galls (Figure 1G) or number of ELFs (Figure 1H), confirming previous observations (Ji et al., 2015). Our data again show a significantly reduced number of galls (Figure 1G) and ELFs (Figure 1H) in AO/DHA-sprayed plants (reduction of 50–70%) when compared with mock-sprayed (Ctrl) plants. Plants sprayed with AO or DHA combined with AOPP, however, show enhanced *M. graminicola* susceptibility, to a level of severity observed in mock-sprayed (Ctrl) plants, suggesting that AO/DHA-induced resistance is dependent on PAL activity.

### Improved Growth and Low Nematode Infection in AO/DHA-Sprayed Plants Is Correlated With Increases in Shoot Fv/Fm, Chlidx, and mARI

**Figure 2:**
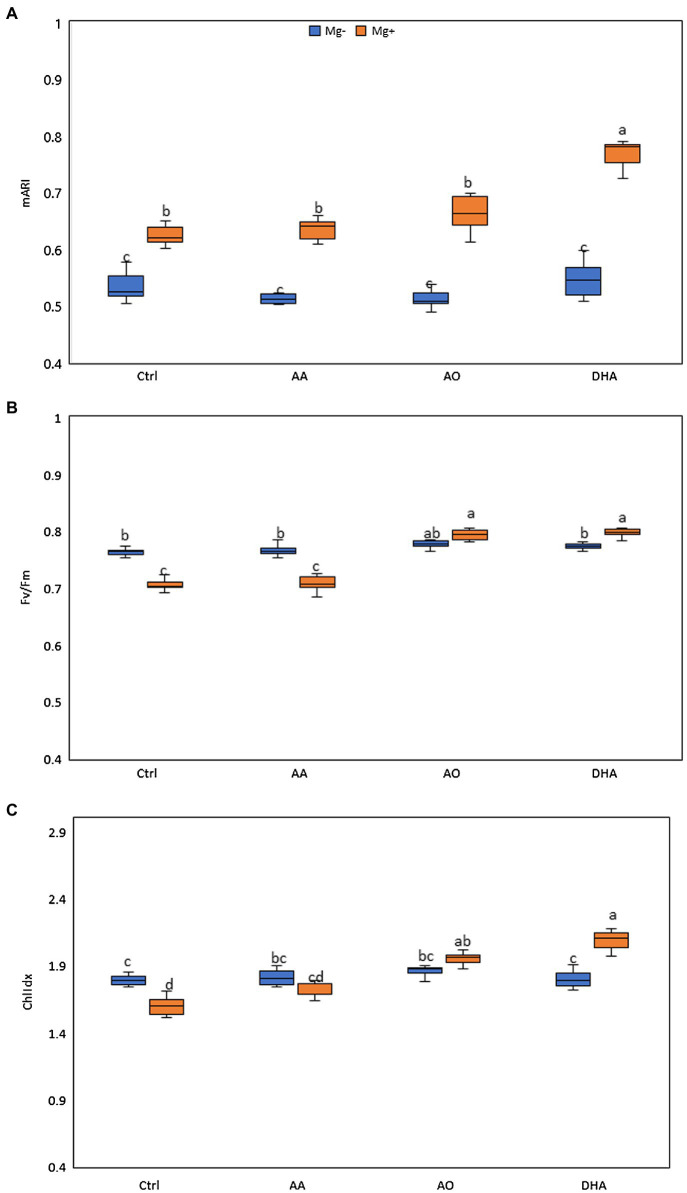
Improved growth and reduced nematode infection in AO/DHA-sprayed plants is correlated with increases in shoot Fv mARI, Fv/Fm, and ChlIdx. The effect of AA (20 mm), AO (20 U/ml), and DHA (20 mm) on **(A)** modified anthocyanin reflectance index (mARI), **(B)** Fv/Fm, and **(C)** ChlIdx in rice leaves. Fourteen-day-old wild-type Nipponbare rice plants were sprayed with 20 mm of AA, 20 ml/unit of AO, and 20 mm of DHA or mock sprayed (Ctrl). After 24 h, plants from each treatment were inoculated with ≈250 s-stage juveniles of *M. graminicola* per plant. Shoots of un/infected plants were monitored for their mARI, Fv/Fm, and ChlIdx values using an automated phenotyping platform (APP) at 3dai (*n* = 5). One-way ANOVA was applied for the statistical analysis (Tukey test, *α* = 0.05), and data are shown by box plots with median. Different letters indicate significant differences among the treatments.

A positive effect of AO/DHA on growth of rice shoots and roots, amid low nematode susceptibility was observed in Supplementary Figure 2. Therefore, in the next experiment, the physiological response of treated plants with or without nematode infection was monitored in detail. Using image-based assessments, we investigated the potential changes in chlorophyll fluorescence imaging (Fv/Fm), ChlIdx, and modified anthocyanin index (mARI). As done before, 14-day-old plants were sprayed and 1 day later, plants were either inoculated with nematodes or mock inoculated. Three days after inoculation, corresponding to 4 days after spraying, the leaves of the plants were monitored using APP. A significantly higher mARI value was generally observed in the shoots of nematode-infected plants when compared with uninfected plants, regardless of the treatment (Figure 2A). Our data further reveal significantly increased mARI values in DHA-sprayed plants upon nematode challenge, when compared with all other treatments (Figure 2A). Looking at the Fv/Fm, the values significantly decreased in shoots of nematode-infected plants when compared with shoots of uninfected plants, but only when plants were mock sprayed or sprayed with AA (Figure 2B). In contrary, the Fv/Fm values in the shoots of AO- or DHA-sprayed infected plants significantly increased when compared with the shoots of mock-sprayed or AA-sprayed plants (Figure 2B).

Confirming the lowered Fv/Fm, the data also show significantly lower values of ChlIdx in shoots of mock-sprayed infected plants when compared with uninfected plants (Figure 2C). Moreover, the ChlIdx values increased significantly in the shoots of AO/DHA-sprayed infected plants when compared to shoots of mock-sprayed uninfected plants. The strongest increase in ChlIdx and Fv/Fm values was observed in DHA-sprayed infected plants. Taken together, nematode infection in untreated plants is correlated with decreases in Fv/Fm and ChlIdx in rice shoot tissue. However, the low nematode infection in AO/DHA-sprayed plants is correlated with increases in Fv/Fm, ChlIdx, and mARI.

### AO/DHA Enhances Root Growth and Induces Tolerance to Nematode Stress

**Figure 3:**
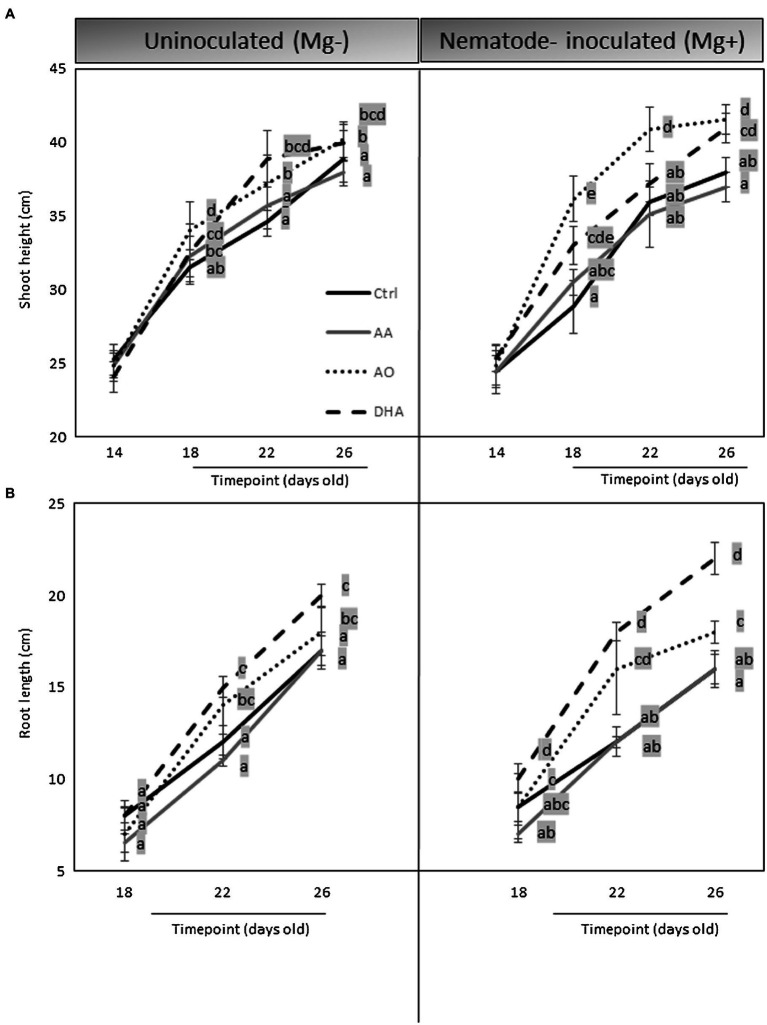
AO/DHA enhances root growth and induces tolerance to nematode stress. Effect of AA, AO/DHA on Nipponbare **(A)** shoots **(B)** root at three time points under uninfected and nematode-infected conditions. Fourteen-days-old rice plants were first measured for their shoot height and then foliar sprayed with AA, AO/DHA, and water as a negative control (Ctrl). One day later, a subset of the plants was inoculated with 250 nematodes per plant (Mg^+^) while a subset was mock inoculated (Mg^−^). Shoots and roots were measured at 18, 22, and 26 days. Data were analyzed by one-way ANOVA followed by Tukey’s *post-hoc* test. Different letters indicate statistically different means at each time point, *p ≤* 0.05.

Because an increased growth and reduced nematode susceptibility were observed in AO/DHA-sprayed plants, a possible induced tolerance phenomenon could also be part of this mechanism. Accurate assessment of plant tolerance to nematodes requires comparative plant growth measurements on plants challenged with nematodes versus control plants (Roberts, 2002) and that is, why we investigated the effect of exogenous applications of AA, AO, or DHA on rice growth under nematode-uninfected and infected conditions. Plants were measured just before spraying (14-days old) and then again measured at 18, 22, and 26 days old with (Mg^+^) or without (Mg^−^) to monitor their growth. A slight decrease (30%, not significant) in shoot height was observed in untreated infected (Mg^+^) plants compared with Ctrl (Mg^−^) at 18 days and 26 days (Figure 3A), which illustrates the mild negative effect of nematode infection on above-ground rice growth. However, this effect was negated when the plants were pre-sprayed with AO or DHA. Interestingly, under nematode infection (Mg^+^), significant increases in shoot height were observed in AO- or DHA-sprayed plants, when compared with untreated infected plants (Mg^+^) at 26 days (Figure 3A). The positive effect of AO/DHA on shoot height was observed as early as 4 days after spraying but was much stronger in nematode-infected plants (Mg^+^) than in uninoculated (Mg^−^) plants. The AO/DHA-treated plants had a significant increase in root length at 22 and 26 days, when compared with untreated or AA-sprayed plants, and this effect seemed magnified mainly under nematode-infected conditions (Figure 3B).

## Discussion

In our previous work, we elucidated the role of ascorbate oxidation in rice defense against *M. graminicola*. In this study, we show that AO/DHA-induced resistance in rice against Mg is correlated with activation of the phenylpropanoid pathway and leads to a tolerant plant phenotype. Apoplastic AO is a blue-copper oxidase that oxidizes AA to DHA (Pignocchi and Foyer, 2003; Foyer and Noctor, 2005; Fotopoulos and Kanellis, 2013; Venkatesh and Park, 2014). AO is involved in a number of signaling cascades (De Tullio et al., 2013), where it influences both symplastic and apoplastic AA/DHA ratios, leading to effects on cell expansion and on the plant growth rate (Esaka et al., 1992; Li et al., 2017) as well as on stress tolerance (Miret and Müller, 2017). Although DHA and AO had clear effects on IR against nematodes, AA application did not activate such phenotype. This could be related to the fact that AA preferentially crosses cellular membranes in the oxidized form – as previously described by (Horemans et al. 1997). However, our previous research confirmed that foliar AA application, at the same concentration and with the same application method as used in the current study, is taken up by the shoots (Singh et al., 2020b). Noteworthy, this previous study also revealed that AA was not accumulating in the roots upon exogenous AA supply, while exogenous AO or DHA application did affect systemic changes in the AA levels as well as its redox state. These observations suggest that the oxidation state of AA would affect its transport in the plant. However, further research is needed to investigate this hypothesis.

An upregulation of *PAL* gene expression and increase in PAL activity was revealed upon application of AO or DHA, with a primed PAL activation in nematode-induced galls of AO/DHA-treated plants. While PAL-activity measurements confirmed enzymatic activation in the shoots of DHA-sprayed plants, its gene expression was negatively affected in this tissue. PAL is known to be metabolically regulated through negative feedback on PAL transcription and enzyme activity by cinnamic acid (Blount et al., 2000). This could indicate that the DHA-induced PAL activity leads to negative feedback control of PAL gene expression in shoot tissue. The phenylpropanoid pathway serves the production of an array of stress-responsive secondary metabolites, which includes SA, flavonoids – among which anthocyanins –, lignin, and hydroxycinnamic acids (Dixon et al., 2002; Desmedt et al., 2020; Yadav et al., 2020). The significance of this pathway in plant defense and induced resistance was previously shown in other crops and against different pathogens. For example, defense priming with BABA was shown to improve soybean resistance against aphids through activation of the phenylpropanoid metabolism and callose deposition (Yao et al., 2020). Similarly, PAL was shown to contribute to the resistance of black rice against *Xanthomonas oryzae* (Solekha et al., 2020).

Induced resistance against nematodes seems generally correlated with activation of the phenylpropanoid pathway. For example in Singh et al. (2019, 2020a), systemic activation of the phenylpropanoid pathway in response to application of COS-OGA in rice or AO in sugar beet was observed to be correlated with induced resistance to parasitic root-knot and cyst nematodes. Similarly, Huang et al. (2016) showed that thiamine-induced resistance against *M. graminicola* in rice involved lignin deposition in plant roots, and this correlated with enhanced transcription of *OsPAL1* and *OsC4H*, two genes involved in the phenylpropanoid pathway. In addition, *OsPAL4* is induced upon infection in the *M. graminicola*-resistant rice cultivar Vandana, while no differential expression was observed in susceptible cultivar Pusa (Kumari et al., 2016). Moreover, Khanam et al. (2018) showed induction of *OsPAL1* and increased PAL activity in the resistant rice cultivar Manikpukha against rice stem nematode *Ditylenchus angustus.* Meta-analysis of transcriptome studies showed increased expression of *OsPAL4* and several other family members during plant resistance to multiple pathogens (Tonnessen et al., 2015). However, chemically blocking PAL by AOPP does not lead to differences in number of galls or number of females in untreated plants, confirming previous observations of Ji et al. (2015). When chemically blocking this enzyme AO and DHA were no longer effective in IR and the plants were equally susceptible as the untreated control plants, showing that the phenylpropanoid pathway is involved in induced resistance against *M. graminicola*. Transcriptome analyses have shown that the phenylpropanoid pathway is generally suppressed in RKN-induced feeding sites in rice (Kyndt et al., 2012). Taken together, these data highlight the role of this pathway in nematode resistance. However, the complexity of the pathway makes it difficult to pinpoint which specific metabolite(s) is/are involved in nematode resistance. Based on the observation that blocking PAL does not enhance susceptibility, it is likely that different metabolites could even play opposite roles in plant susceptibility or defense. Our recent research revealed that temporary perturbation of the phenylpropanoid pathway leads to a general reprogramming of the plant defense metabolome, and broad-spectrum-induced resistance in tomato (Desmedt et al., 2021). Which metabolite(s) is/are responsible for this IR remains to be elucidated.

In addition to directly activating the plant’s immune system, some IR stimuli can trigger priming of specific defense genes or pathways, a phenomenon which was also observed here in our research. Defense priming is an adaptive part of induced resistance, that is, marked by an enhanced activation of defense mechanisms upon pathogen attack. Upon initial stimulus perception – e.g., ascorbate oxidation – changes may occur in the plant at the physiological, transcriptional, metabolic, and epigenetic levels. This phase is called the priming phase and upon subsequent challenge, the plant effectively mounts a faster and/or stronger defense response, resulting in increased resistance and/or improved stress tolerance (Mauch-Mani et al., 2017; De Kesel et al., 2021). Some IR stimuli have been shown to lead to fitness costs, and as argued by Martinez-Medina et al. (2016), this should be evaluated for every stimulus. Our data rather revealed a positive effect of AO and DHA on rice growth, with increased rice shoot and root growth. These results clearly illustrate how IR is not generally accompanied with fitness costs.

Flavonoids constitute a large class of phenylpropanoid-derived carbon-based metabolites present in all land plants. There are several flavonoid subgroups based on their structural properties, including the chalcones, flavones, flavonols, flavandiols, condensed tannins, isoflavonoids, and anthocyanins (Winkel-Shirley, 2001; Erb and Kliebenstein, 2020). The spectral parameter mARI was found to be a good proxy for anthocyanin content in monocots and dicots (Gitelson et al., 2009). The mARI data suggest that anthocyanins are accumulating in leaves of AO/DHA-stimulated rice plants. A defense response in plants is often characterized by accumulation of foliar anthocyanins, which are produced as antioxidants (Steyn et al., 2002; Gould and Lister, 2006) to cope with the plant’s reactive oxygen species ROS burst (Xu et al., 2017). Anthocyanins are known to have ROS scavenging properties and hence protect the cells from oxidative damage (Landi et al., 2015; Xu et al., 2017). Our image-based mARI data showed that nematode infection causes accumulation of anthocyanins in the shoots, correlating with the reported above-ground oxidative response upon RKN infection (Kyndt et al., 2017). Increased H_2_O_2_ levels were also previously reported upon AO treatment (Singh et al., 2020b). DHA/AO-sprayed plants showed a primed defense response against nematodes correlated with potentiated mARI values. Next to that, our image-based data show that low nematode infection in AO/DHA-sprayed plants is correlated with increases in Fv/Fm and ChlIdx. Similarly, Fan et al. (2020) showed that COS derivatives chitosan-thiadiazole-trifluorobutene (COSSZFB) activate induced resistance against *M. incognita* in cucumber and simultaneously improved plant growth through accumulation of photosynthetic pigments. Fv/Fm values are known to decrease along with the increasing effect of stresses (Rolfe and Scholes, 2010; Gorbe and Calatayud, 2012). For example, Meng et al. (2020) showed stronger reduction in Fv/Fm values in strawberry leaves with high infection of Botrytis cinerea similar to what we observe in leaves of untreated, nematode-infected plants.

DHA/AO-IR against RKN in rice was previously shown to result in a primed jasmonate response, the major pathway involved in rice defense against RKN (Nahar et al., 2011). Most likely, a systemic signaling cascade based on redox signals and/or DHA is activated upon foliar AO treatment. Likewise, a shoot-root signaling pathway, integrating ROS, and JA were shown to be involved in tomato defense against RKN (Wang et al., 2019). Noteworthy, it is known that the phenylpropanoid pathway is positively regulated by JA (Pauwels et al., 2008; Taheri and Tarighi, 2010).

The significant increases in chlorophyll fluorescence and growth of nematode-infected rice plants pre-sprayed with AO/DHA implies that ascorbate oxidation induces a tolerance mechanism. Plant tolerance is a phenotype where the negative effect of pathogens is low, and plant growth and yield are not negatively affected (Koch et al., 2016). Tolerance to nematode stress involves compensation *via* the growth of new tissues, increased number of roots, and enhanced water and nutrient uptake (Franco and Evans, 1979; Trudgill and Cotes, 1983; Fatemy and Evans, 1986a,b; Miltner et al., 1991). For example, Trudgill and Cotes (1983) showed that potato cultivars that are tolerant to cyst nematodes *G. rostochiensis* have a higher number of roots and increased root weight when under attack by nematodes. Similarly, Swain and Prasad (1988) showed increased chlorophyll content as a mechanism of tolerance of rice to *M. graminícola*. To confirm that these plants are really tolerant to nematode infestation, field trials in natural nematode-infested soils will be required.

Enhancing the natural tolerance/defense of plants toward different stress factors provides a durable approach for enhanced and sustainable crop production. Tolerance combined with resistance is preferred over tolerance alone because large healthy roots of tolerant, susceptible plants allow the nematode population to increase (Roberts, 2002). Our research unveils that next to inducing resistance, ascorbate oxidation also positively affects plant tolerance to nematodes in rice. AO and DHA are therefore promising compounds to be used in sustainable crop protection.

## Data Availability Statement

The original contributions presented in the study are included in the article/Supplementary Material, and further inquiries can be directed to the corresponding author.

## Author Contributions

TK and RS planned and designed the research and wrote the manuscript. RS, KA, and JP conducted phenotyping and infection experiments. RS conducted biochemical analysis. All authors read and approved the manuscript.

## Conflict of Interest

A patent about the use of DHA to protect plants from nematode infection has been submitted by Ghent University. The inventors are authors RS and TK.

The remaining authors declare that the research was conducted in the absence of any commercial or financial relationships that could be construed as a potential conflict of interest.

## Publisher’s Note

All claims expressed in this article are solely those of the authors and do not necessarily represent those of their affiliated organizations, or those of the publisher, the editors and the reviewers. Any product that may be evaluated in this article, or claim that may be made by its manufacturer, is not guaranteed or endorsed by the publisher.
