# Supplementary material for: Induced Resistance by Ascorbate Oxidation Involves Potentiating of the Phenylpropanoid Pathway and Improved Rice Tolerance to Parasitic Nematodes
Source: Front Plant Sci. 2021 Aug 11;12:713870. doi: 10.3389/fpls.2021.713870 (PMC8386471; doi:10.3389/fpls.2021.713870)
Supplement: Supplementary file 1 [file Presentation_1.PPTX]

## Slide 1
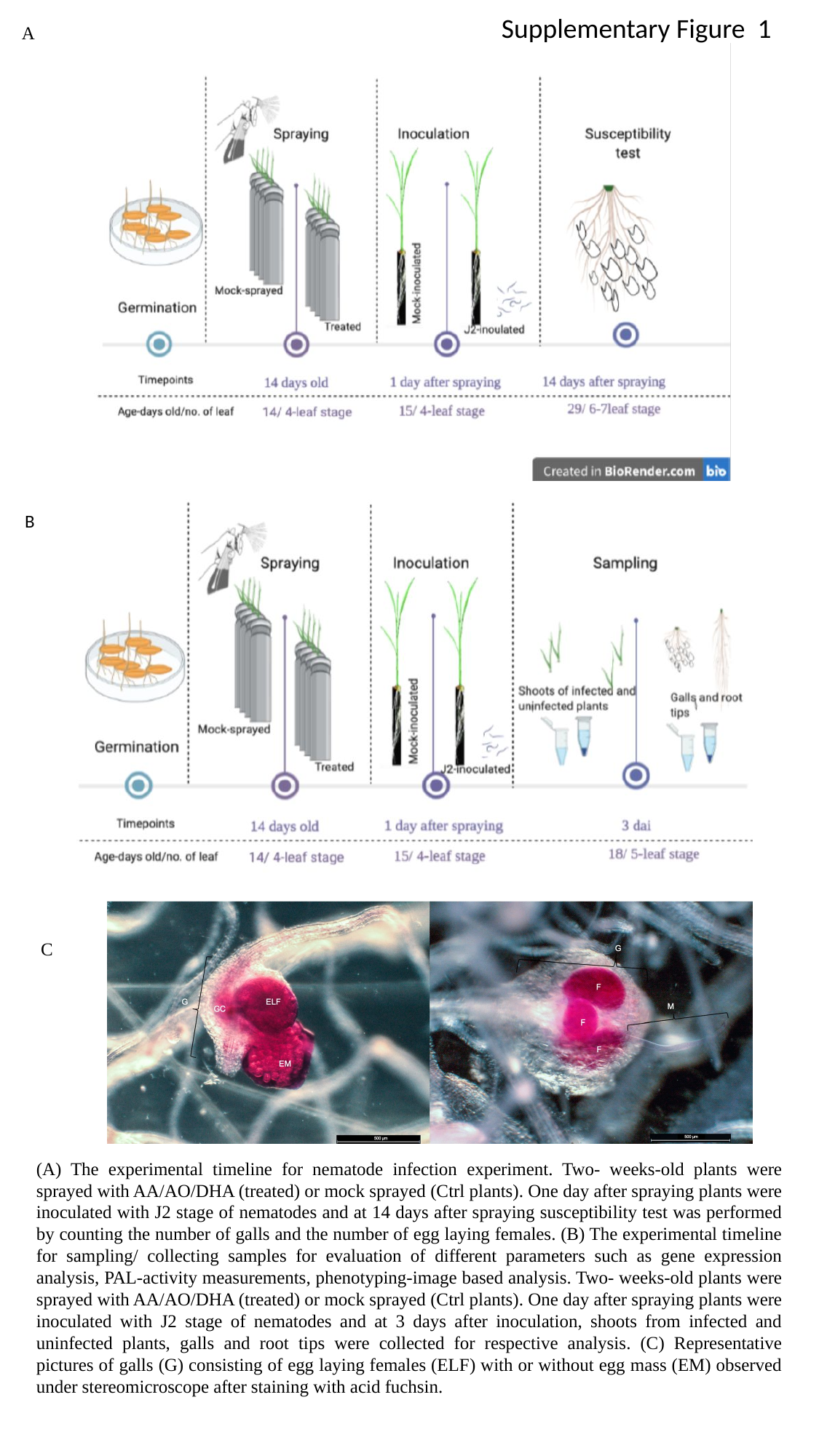

Supplementary Figure 1
A
B
C
(A) The experimental timeline for nematode infection experiment. Two- weeks-old plants were sprayed with AA/AO/DHA (treated) or mock sprayed (Ctrl plants). One day after spraying plants were inoculated with J2 stage of nematodes and at 14 days after spraying susceptibility test was performed by counting the number of galls and the number of egg laying females. (B) The experimental timeline for sampling/ collecting samples for evaluation of different parameters such as gene expression analysis, PAL-activity measurements, phenotyping-image based analysis. Two- weeks-old plants were sprayed with AA/AO/DHA (treated) or mock sprayed (Ctrl plants). One day after spraying plants were inoculated with J2 stage of nematodes and at 3 days after inoculation, shoots from infected and uninfected plants, galls and root tips were collected for respective analysis. (C) Representative pictures of galls (G) consisting of egg laying females (ELF) with or without egg mass (EM) observed under stereomicroscope after staining with acid fuchsin.

## Slide 2
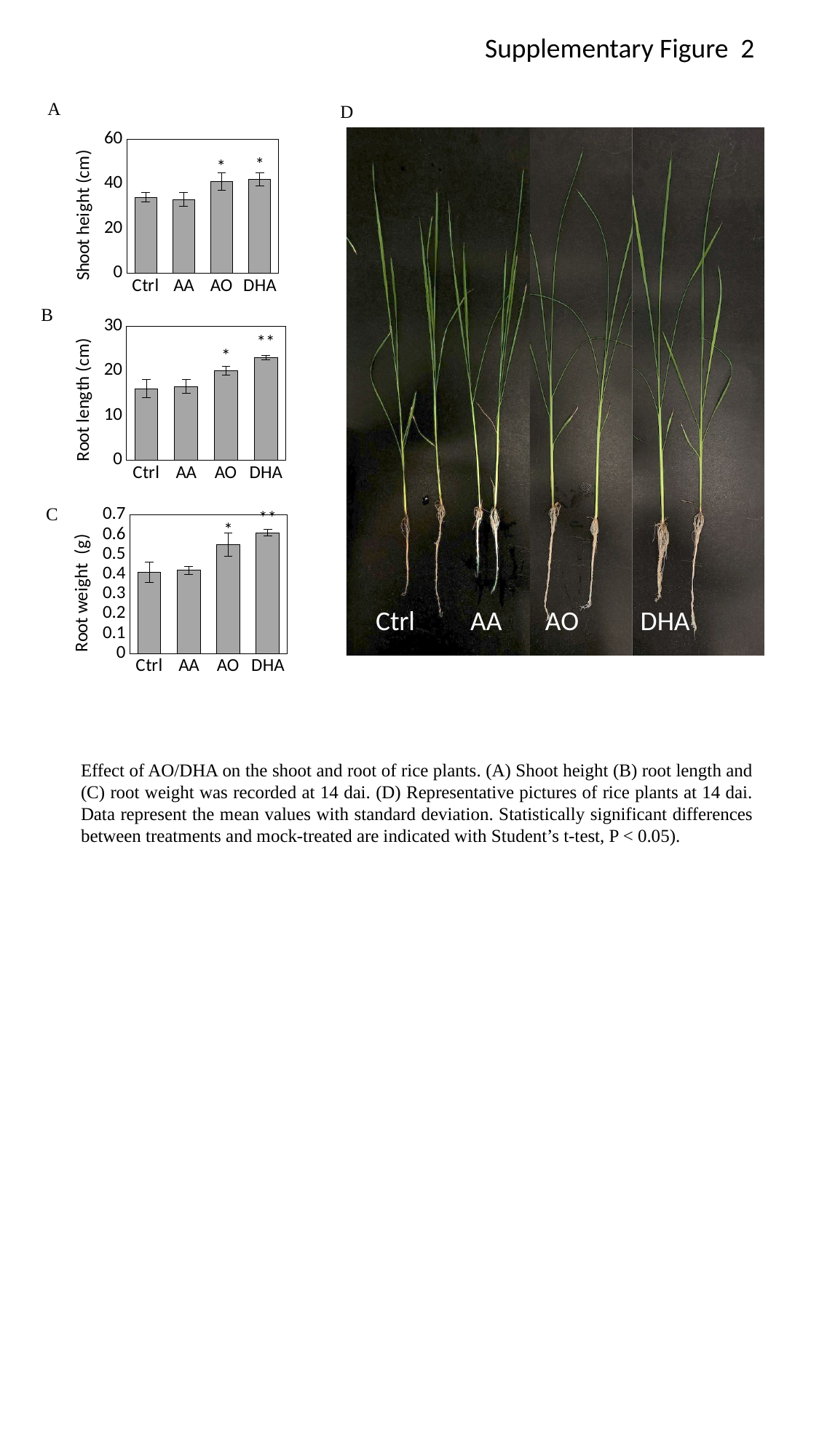

Supplementary Figure 2
A
### Chart
| Category | SH |
|---|---|
| Ctrl | 34.0 |
| AA | 33.0 |
| AO | 41.0 |
| DHA | 42.0 |D
 Ctrl AA AO DHA
B
### Chart
| Category | RL |
|---|---|
| Ctrl | 16.0 |
| AA | 16.5 |
| AO | 20.0 |
| DHA | 23.0 |C
### Chart
| Category | RW |
|---|---|
| Ctrl | 0.41 |
| AA | 0.42 |
| AO | 0.55 |
| DHA | 0.61 |Effect of AO/DHA on the shoot and root of rice plants. (A) Shoot height (B) root length and (C) root weight was recorded at 14 dai. (D) Representative pictures of rice plants at 14 dai. Data represent the mean values with standard deviation. Statistically significant differences between treatments and mock-treated are indicated with Student’s t-test, P < 0.05).
